# On Prolapsus of the Uvula into the Rima Glottidis

**Published:** 1830-10-01

**Authors:** 


### XLII.

### PROLAPSUS OF THE UVULA.

WE have reason to believe that some cases of sudden death in fevers, and especially in scarlatina anginosa, are occasioned by the introduction of the elongated and relaxed uvula into the rima glottidis, when the patient is asleep, and thus proving immediately fatal. The following remarks of Baron Larrey may not prove useless if borne in mind.

"In certain diseases," says he, "especially in angina, low fevers, and syphilis, the uvula becomes engorged and relaxed to such a degree that it falls on the glottis and keeps up a constant irritation there, sometimes occasioning actual suffocation, if not promptly relieved. In these circumstances all the usual means fail, and nothing but excision of the uvula succeeds. This is best done by seizing the point of the uvula with a pair of forceps, and snipping it off with a pair of scissors. The part should not be cut off too near the base of the uvula. A young Portuguese merchant (M. Teyxera) in the ninth day of an ataxic fever, was seized with angina gangrenosa, the uvula being the first part affected, and projecting into the rima glottidis and threatening instant suffocation. The voice was extinguished, and all deglutition rendered impossible. The Baron was called to him in the middle of the night, and on examining the throat, he found the uvula drawn down into the rima glottidis, to the extent of nearly half an inch, by the act of inspiration. It was black, and appeared to drop off and go down into the larynx. He had considerable difficulty in disengaging the part from the cavity into which it had been sucked, and then cut it away. The relief was immediate—respiration went on freely—and this young gentleman was snatched from impending asphyxia." We repeat our conviction, from some instances in our own practice and in that of others, that sudden death sometimes arises from the above cause in fevers and other acute diseases.

